# Bosutinib for Successful Treatment‐Free Remission in Chronic Myeloid Leukemia

**DOI:** 10.1002/cam4.70822

**Published:** 2025-04-28

**Authors:** Yuki Fujioka, Maiko Abumiya, Takaaki Ono, Naoto Takahashi

**Affiliations:** ^1^ Department of Hematology, Nephrology and Rheumatology Akita University Graduate School of Medicine Akita Japan; ^2^ Division of Central Laboratory Akita University Hospital Akita Japan; ^3^ Department of Pharmacy Akita University Hospital Akita Japan; ^4^ Division of Transfusion and Cell Therapy Hamamatsu University Hospital Hamamatsu Japan

**Keywords:** bosutinib, chronic myeloid leukemia, effector memory CD8^+^ T cells, effector regulatory T cell, treatment‐free remission

## Abstract

**Introduction and Objective:**

This study explored the status of patients with chronic myeloid leukemia following the safe discontinuation of frontline or second‐line bosutinib treatment. The goal was to assess the long‐term outcomes and factors influencing treatment‐free remission (TFR) following cessation of bosutinib therapy.

**Methods:**

The median duration of bosutinib treatment among 16 patients was 48 months. All patients achieved a deep molecular response before bosutinib discontinuation, which was sustained for a median pre‐cessation period of 27 months. Patients were monitored for molecular response and clinical outcomes.

**Results:**

After bosutinib discontinuation, the major molecular response was lost in six patients: within 6 months in five patients and at 19 months in one patient. All six patients achieved a major molecular response after at least 3 months of bosutinib re‐treatment. Ten patients exhibited successful TFR without loss of major molecular response for a median duration of 48 (16–101) months. Kaplan–Meier analysis revealed a 68.8% treatment‐free survival at 12 months. After bosutinib cessation, eight patients developed Grade 1–2 withdrawal syndrome. No differences were observed in the clinical characteristics or bosutinib treatment between patients with TFR at 12 months (TFR group) and those without remission (recurrence group), except for the deep molecular response duration before bosutinib cessation (31 vs. 24 months, *p* = 0.009). T‐cell profiling using flow cytometry revealed a higher percentage of effector memory CD8^+^ T cells at 1 and 3 months after bosutinib discontinuation in the TFR group than in the recurrence group (*p* = 0.012 and *p* = 0.005, respectively).

**Conclusion:**

Bosutinib can be safely discontinued under certain conditions, similar to other tyrosine kinase inhibitors. Additionally, T‐cell profile analysis before and after bosutinib discontinuation may predict successful TFR.

## 1 Introduction

BCR:ABL1 tyrosine kinase inhibitors (TKIs) improve the long‐term life expectancy of patients with chronic myeloid leukemia (CML), similar to that of age‐matched populations [1]. Following the Stop Imatinib trial—a trial on the discontinuation of the first‐generation TKI imatinib in 2010 [2]—several trials on TKI discontinuation, including those involving the second‐generation TKIs nilotinib and dasatinib, have reported no relapse in some patients after long‐term molecular remission following TKI discontinuation [3, 4, 5, 6, 7]. Consequently, treatment‐free remission (TFR) has emerged as a new therapeutic goal for TKI therapy in patients with CML [8].

The National Comprehensive Cancer Network Guideline version 1.2023 and JSH Practical Guidelines for Hematological Malignancies 2023 outline the following criteria for TFR: adult patients with chronic CML who have not experienced accelerated phase (AP) or blast crisis (bc), receiving TKI therapy for ≥ 3 years, and sustained deep molecular response (DMR) for ≥ 2 years. Additionally, regular monitoring of *BCR::ABL1* mRNA levels using high‐sensitivity polymerase chain reaction (PCR) following TKI discontinuation is essential, along with prompt resumption of treatment upon loss of the major molecular response (MMR) [9, 10].

The European LeukemiaNet (ELN) recommendations are slightly more stringent, requiring at least 5 years of TKI treatment, if possible. The minimum requirement is at least 4 years for second‐generation TKIs and first‐ or second‐line therapy if intolerance is the only reason for changing TKI. Thus, resistant cases switched using the ELN criteria were excluded from the TFR recommendation [11].

Although immune responses to TKI discontinuation have been extensively reported, a unified view is yet to be established [12, 13, 14, 15]. In a previous report, we examined immune responses to imatinib discontinuation and detected a transient increase in effector regulatory T cells (eTregs) [16]. Activation of the immune response may be necessary to maintain TFR.

Bosutinib is an ATP‐competitive BCR:ABL1 TKI that exhibits Src family kinase inhibitory activity in imatinib‐resistant BCR:ABL1 point‐mutant cells, except for T315I and V299L. The BFORE Trial, a multinational phase III study involving patients with newly diagnosed chronic‐phase CML, reported a statistically significant higher and faster MMR in bosutinib‐treated patients than in imatinib‐treated patients after a 5‐year follow‐up period [17]. In Japan, bosutinib was approved in 2014 for CML that was resistant or intolerant to prior TKIs and in 2020 for newly diagnosed chronic‐phase CML [18, 19, 20, 21]. However, given that it was launched after other second‐generation TKIs, such as nilotinib and dasatinib, reports of TFR using bosutinib remain limited.

Therefore, in this study, we aimed to explore the clinical status of patients with CML following the safe discontinuation of bosutinib therapy.

## 2 Methods

### 2.1 Study Design and Patients

This study included patients with chronic‐phase CML who received bosutinib treatment at Akita University Hospital and Hamamatsu University Hospital between July 2009 and August 2022. This study was conducted in accordance with the principles of the Declaration of Helsinki and was approved by the Ethics Committee of the Akita University Graduate School of Medicine (no. 2235). All participants provided informed consent and signed an institutional review board consent form.

Bosutinib was administered as first‐ or second‐line therapy because of an inadequate response or intolerance to prior TKI therapy. An insufficient response was defined as achieving MMR but not DMR after at least 18 months of treatment.

Bosutinib was discontinued in patients who were motivated to discontinue TKIs for any reason, received TKI treatment for ≥ 3 years (including prior TKI treatment), and exhibited sustained MR4.5 (*BCR::ABL1* international scale < 0.0032%, 4.5‐log reduction of *BCR::ABL1* mRNA) for ≥ 2 years, confirmed using four consecutive PCR tests. Despite insufficient treatment duration (21 months), frontline bosutinib was discontinued in one patient because of chemotherapy for breast cancer.

**FIGURE 1:**
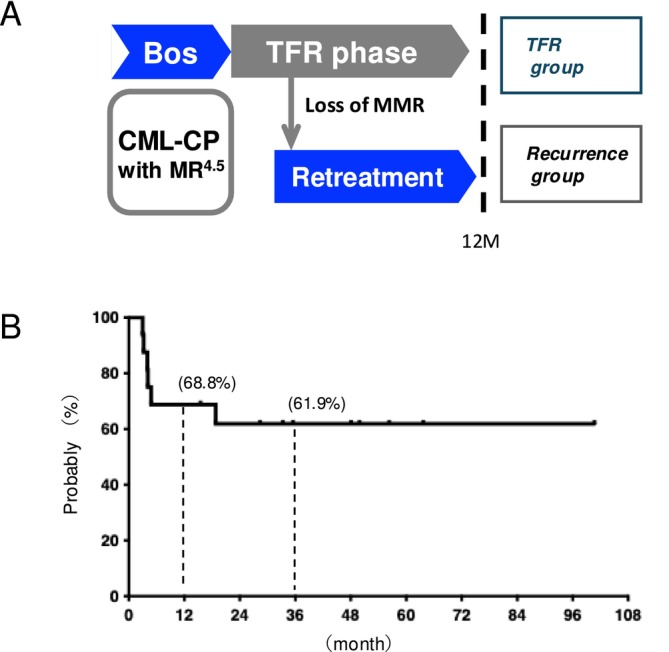
TFS in patients treated with bosutinib. (A) Schematic summary of the study. TFR was defined as no loss of MMR (IS < 0.1%) following bosutinib discontinuation. Molecular responses were assessed regularly using IS‐PCR in the TFR phase. Re‐treatment was initiated immediately at the loss of MMR. Patients were classified into two groups according to the TFR at 12 months to compare their characteristics and immunological changes. (B) Kaplan–Meier analysis of TFS following bosutinib discontinuation. TFS was defined as the time from bosutinib discontinuation to the loss of MMR. For patients who achieved TFR, survival was censored at the date of the last assessment. Bos, bosutinib; CML‐CP, chronic myeloid leukemia chronic phase; MMR, major molecular response; MR4.5, 4.5‐log reduction in *BCR::ABL1* mRNA; TFR, treatment‐free remission.

Plasma bosutinib concentrations (trough plasma concentration, *C*_0_) were measured using high‐performance liquid chromatography as described previously [22]. Peripheral blood samples were collected before bosutinib discontinuation and 1, 3, 6, and 12 months after discontinuation (Figure 1A). Peripheral blood mononuclear cells were isolated and analyzed for immunophenotypes using flow cytometry as described previously [16].

### 2.2 Statistical Analysis

Statistical analyses were conducted using SPSS software version 28.0 Windows (IBM Corp., Armonk, NY, USA). The clinical characteristics of patients are expressed as numbers or medians (range: minimum–maximum). The daily dose, *C*_0_ of bosutinib, and treatment duration are expressed as medians and ranges. Nonparametric variables were compared between the groups using the Wilcoxon signed‐rank test, Mann–Whitney *U* test, or Fisher's exact test. TFR duration was measured from the date of bosutinib discontinuation to the date of bosutinib readministration due to loss of MMR or the date of the last molecular examination for patients with no relapse. Kaplan–Meier analysis was used to estimate treatment‐free survival (TFS). Statistical significance was set at *p <* 0.05.

## 3 Results

### 3.1 Patients

This study included 16 patients with median ages of 48 (32–72) and 58 (36–79) years at CML diagnosis and bosutinib discontinuation, respectively. The male:female ratio was 1:1. All patients had chronic‐phase CML without a history of AP/BC. Based on the Sokal scores, three, five, and seven patients were classified as high‐, intermediate‐, and low‐risk, respectively; one patient could not be evaluated. No additional chromosomal abnormalities were detected in the 14 patients with available karyotype data. Nine patients received bosutinib therapy as the first‐line treatment for newly diagnosed CML, whereas seven received bosutinib as the second‐line treatment for CML due to intolerance (three patients with imatinib and two with dasatinib) or an insufficient response (two patients with MMR to nilotinib) to prior TKIs. The median duration of TKI treatment was 52 (21–165) months.

### 3.2 Bosutinib Treatment and TFR After Its Discontinuation

The median duration of bosutinib treatment was 48 (16–154) months, and the median bosutinib dose was 350 (200–500) mg. Notably, two patients with liver dysfunction received a lower bosutinib dose of 200 mg. The median *C*_0_ of bosutinib was 75.6 (49–116.0) ng/mL. All patients achieved MR4.5 before bosutinib discontinuation. The median pre‐cessation duration of MR4.5 was 27 (16–129) months. Loss of MMR was detected within 6 months in five patients and at 19 months in one patient following bosutinib discontinuation; however, it did not lead to AP/bc progression in any patient. These patients achieved MMR within 3 months of bosutinib re‐treatment. The remaining 10 patients (62.5%) achieved successful TFR without MMR loss after bosutinib discontinuation for a median duration of 48 (range, 16–101) months. The median observation period following TKI discontinuation was 31 (range, 2–101) months. Kaplan–Meier analysis revealed TFS rates of 68.8% and 61.9% at 12 and 36 months, respectively (Figure 1B). Eight patients developed Grade 1–2 withdrawal syndrome (WS) following bosutinib discontinuation.

Univariate analysis revealed no significant association between the TFR and patient characteristics, including age, sex, Sokal risk score, prior TKI treatment, TKI treatment duration, bosutinib dose, *C*_0_ of bosutinib, bosutinib treatment duration, and WS following bosutinib discontinuation (Table 1). However, the median DMR duration was significantly longer in patients who achieved TFR than in those who did not (31 [24–129] vs. 24 [16–27] months, *p* = 0.009, Mann–Whitney *U* test).

**TABLE 1:** Comparison of patients' characteristics between TFR group and recurrence group.

	TFR group	Rec. group	*p*
Patients number	11	5	
Age at TFR (years)	51 (43–79)	67 (36–76)	0.661
Gender, male/female	6/5	2/3	0.500
Sokal risk, low + int/high	7/3	5/0	0.264
Prior TKI, yes/no	6/5	1/4	0.231
TKI duration (months)	80 (36–165)	48 (21–53)	0.115
Bos duration (months)	50 (16–154)	48 (21–53)	0.320
Bos daily dose (mg/day)	400 (200–500)	300 (200–400)	0.377
Trough conc. (ng/mL)	83.6 (49.0–116.0)	72.0 (53.1–75.6)	0.287
DMR duration (months)	31 (24–129)	24 (16–27)	0.009
UMRD at stop TKI, yes/no	10/1	4/1	0.542
WS, Grade 0/1–2	5/6	3/2	0.500

Abbreviations: Bos, bosutinib; conc., concentration; DMR, deep molecular response; Rec., recurrence; TFR, treatment‐free remission; TKI, tyrosine kinase inhibitor; UMRD, undetectable molecular residual disease; WS, withdrawal syndrome.

### 3.3 Immunophenotype Alterations in T Cells Before and After Bosutinib TFR

**FIGURE 2:**
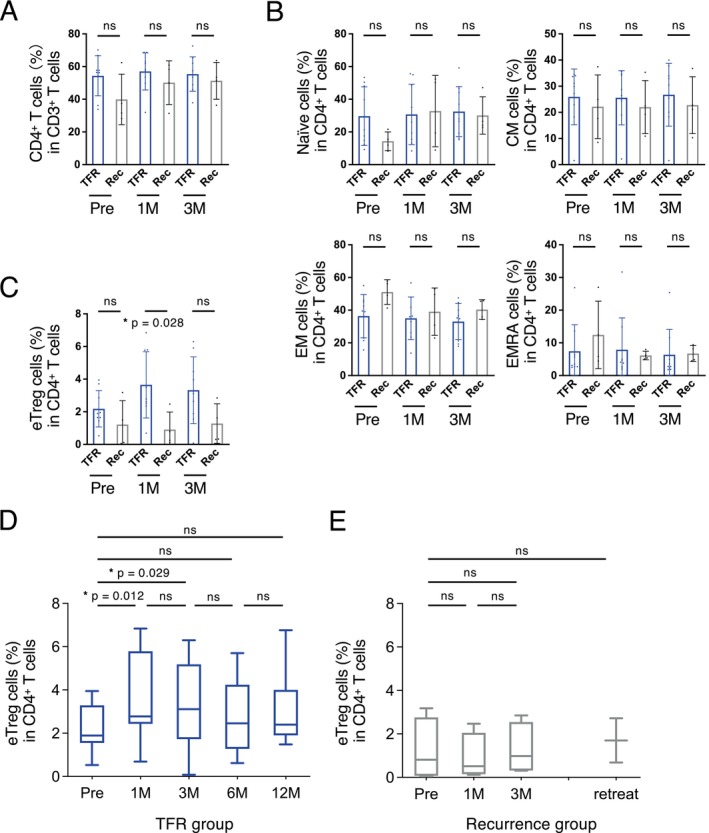
Proportion and subsets of CD4^+^ T cells. (A) Proportion of CD4^+^ T cells and (B) subsets of CD4^+^ T cells (naïve, CM, EM, and EMRA cells) before discontinuation (Pre), at 1 month (1 M), and 3 months (3 M) are presented. No differences can be observed in the proportions of CD4^+^ T cells and subsets of each group between the TFR (blue) and recurrence (gray) groups. (C) eTregs at pre, 1 M, and 3 M are presented. A difference can be observed in the proportions of eTregs between the TFR (blue) and recurrence (gray) groups at 1 M (*p* = 0.028). Kinetics of eTregs from patients with CML in the (D) TFR and (E) recurrence groups. The proportion of eTregs at 1 M or 3 M increases significantly compared with those before discontinuation in the TFR group (*p* = 0.012, *p* = 0.029). However, no significant difference can be observed in the proportions of eTregs at 1 M or 3 M compared with those at pre in the recurrence group. 1 M, 1 month after bosutinib discontinuation; 3 M, 3 months after bosutinib discontinuation; CM cells, central memory cells; EM cells, effector memory cells; EMRA cells, CD45RA^+^ effector memory cells; eTregs, effector regulatory T cells; * *p* < 0.05; ns, not significant; TFR, treatment‐free remission.

**FIGURE 3:**
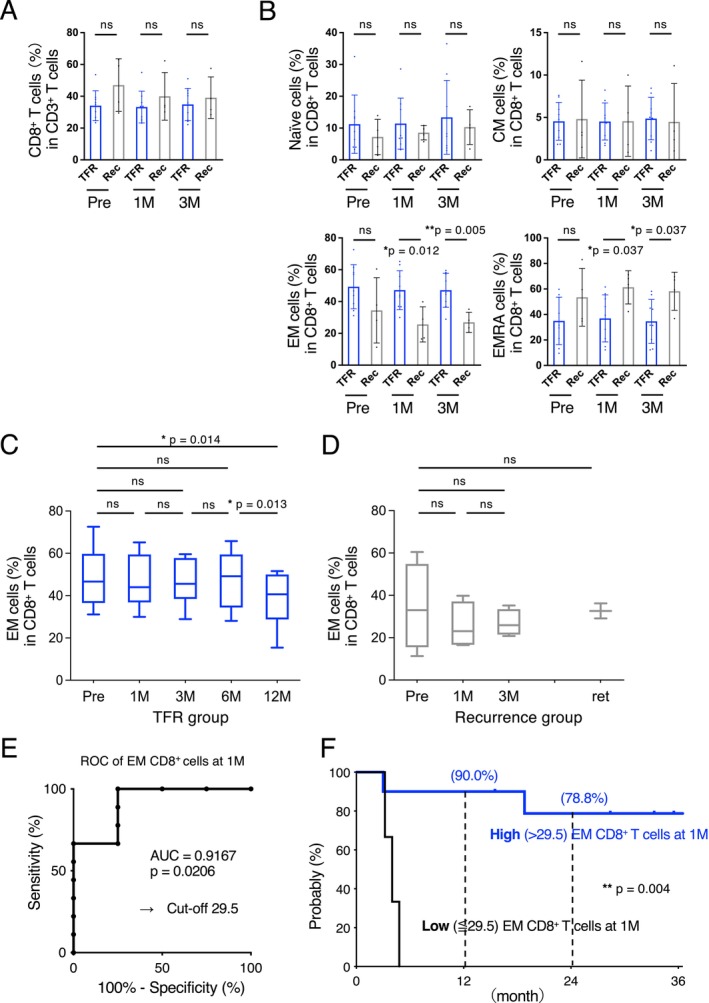
Proportion and subsets of CD8^+^ T cell. (A) Proportion of CD8^+^ T cells and (B) subsets of CD8^+^ T cells (naïve cells, CM cells, EM cells, and EMRA cells) before discontinuation (Pre), at 1 month (1 M), and 3 months (3 M) are presented. Significant differences can be observed in the proportions of the EM cells between the TFR (blue) and recurrence (gray) groups (*p* = 0.012, *p* = 0.005). Kinetics of EM CD8^+^ T cells from patients with CML in the (C) TFR and (D) recurrence groups. (E) The ROC curve shows a 29.5% cutoff value for EM CD8^+^ T cells predicting TFR, calculated using the Youden index. (F) A significant difference can be observed in TFS between patients with high EM CD8^+^ T cells and low EM CD8^+^ T cells using the log‐rank test. 1 M, 1 month after bosutinib discontinuation; 3 M, 3 months after bosutinib discontinuation; AUC, area under the curve; CM, central memory cells; EM, effector memory cells; EMRA, CD45RA^+^ effector memory cells; ns, not significant; Pre, immediately before bosutinib discontinuation; Rec, recurrence; ROC, receiver operating characteristic curve; TFR, treatment‐free remission; * *p* < 0.05; ** *p* < 0.005.

A comparison between the successful TFR and MMR loss groups revealed no differences in CD4^+^ (Figure 2A,B) or CD8^+^ T cells (Figure 3A). Furthermore, we detected no difference in the number of CD19^+^ B cells, CD16^+^ NK cells, or myeloid‐derived suppressor cells (Figure S1).

One month after bosutinib discontinuation, the TFR group had a higher proportion of FoxP3^hi^CD45RA^−^ eTregs than the recurrence group (*p* = 0.028; Figure 2C). However, eTregs in the TFR group increased 1 and 3 months after bosutinib discontinuation compared with the proportions before discontinuation (*p* = 0.012 and *p* = 0.029, respectively; Figure 2D); however, no such changes were observed in the recurrence group (Figure 2E).

Additionally, the TFR group exhibited higher proportions of effector memory (EM) and CD45RA^+^ EM (EMRA) CD8^+^ T cells 1 and 3 months after bosutinib discontinuation than the recurrence group (*p* = 0.012 and 0.005 in EM, *p* = 0.037 and 0.037, respectively; Figure 3B). Notably, this increase in EM CD8^+^ T cells in the TFR group persisted for at least 6 months (Figure 3C).

Receiver operating characteristic curve analysis and Youden index criteria were used to estimate the cutoff value for EM CD8^+^ T‐cell proportions (area under the curve = 0.9167, *p* = 0.0206; Figure 3E). Based on the proportion of EM CD8^+^ T cells (29.5%), the Kaplan–Meier curve revealed a significant difference in TFS between the two groups (*p* = 0.004; Figure 3F).

## 4 Discussion

In this study, our findings indicated that TFR can be achieved using bosutinib therapy, similar to previously reported TKIs, under certain conditions. However, TFR could not be achieved in one patient following frontline bosutinib treatment for < 3 years (21 months), given that bosutinib was discontinued because of breast cancer treatment. The patient had a DMR duration of < 24 months (16 months). As indicated in the guidelines [9, 10, 11], TKI treatment for at least 3 years and a 2‐year DMR are essential criteria for TFR.

Despite the small sample size, this study revealed a significant difference in DMR duration based on patient characteristics between the TFR and recurrence groups. The DMR duration is an important factor for successful TFR, as reported in the EURO‐SKI study, the largest prospective study [23]. Moreover, the DMR duration was a critical clinical factor for TFR following bosutinib treatment in our study. Maintaining at least 2 years of DMR, as indicated in the guidelines, is a prerequisite for initiating TFR, and maintaining a longer DMR could be beneficial for achieving a higher TFR success rate.

Achieving TFR with imatinib therapy is difficult in patients with high‐risk Sokal scores [2]; however, bosutinib therapy achieved successful TFR, even in patients with high‐risk Sokal scores. Nevertheless, our study did not include treatment‐resistant cases based on the ELN criteria. Therefore, challenges related to achieving TFR remain unresolved because DMR, a necessary condition for TFR, is difficult to achieve in TKI‐resistant cases. In this study, all seven patients who switched from a previous TKI to bosutinib achieved DMR, except for one patient who originally achieved DMR. Furthermore, six of the seven patients also achieved successful TFR, of which two exhibited molecular remission resistance to nilotinib, three exhibited imatinib intolerance and resistance, and one exhibited dasatinib intolerance. In general, switching to another second‐generation TKI is ineffective in patients who are resistant to second‐generation TKI [11, 24]. However, switching to bosutinib may achieve DMR in such patients and in those who do not achieve DMR after several years of second‐generation TKI therapy.

The ELN guidelines also recommend switching to 2G‐TKI if TFR is achieved and if DMR is not achieved in patients with a high priority for TFR [11]. The BFORE trial reported a faster and higher rate of DMR achievement with bosutinib than with imatinib in patients with newly diagnosed chronic‐phase CML [17]. Furthermore, switching to bosutinib can help achieve a molecular response in patients who cannot achieve an adequate therapeutic effect owing to resistance or intolerance to imatinib and other 2G‐TKIs [18, 19, 25]. Our results revealed that switching to bosutinib may help achieve successful TFR when achieving DMR is difficult in patients with a high priority for TFR.

The standard bosutinib dose was 400 mg QD for newly diagnosed CML and 500 mg QD for pre‐TKI‐resistant and pre‐TKI‐intolerant CML. In the present study, the bosutinib dose reduction was performed in six patients owing to side effects, such as hepatotoxicity and diarrhea, to ensure long‐term bosutinib treatment. Notably, the daily bosutinib dose was not associated with TFR failure. The median bosutinib doses were 400 (200–500) and 300 (200–400) mg/day in patients with and without TFR at 12 months, respectively (*p* = 0.377, Mann–Whitney *U* test). Even at low doses, the *C*_0_ of bosutinib did not differ significantly from that at the standard dose. No association was observed between *C*_0_ and the bosutinib daily dose (*p* = 0.923, Spearman's rank correlation coefficient test). The BFORE study reported a higher bosutinib *C*_0_ in patients who achieved MMR by 18 months than in those who did not achieve MMR at any observation point (Days 28, 56, and 84). The median *C*_0_ values in patients with and without MMR by 18 months were 94.4 (20.4–300.0) and 53.6 (0.5–97.7) ng/mL in Asian patients and 69.7 (10.8–257.5) and 53.5 (2.3–453.0) ng/mL in non‐Asian patients, respectively [26]. Individual differences in bosutinib pharmacokinetics can be attributed to differences in blood bosutinib concentrations at the same dose [27]. Although bosutinib blood concentrations in this study were maintained at sufficient levels (≥ 70 ng/mL) during the stable state to ensure a therapeutic effect in most patients, no significant difference in *C*_0_ of bosutinib was detected between the TFR and recurrence groups.

Immunological analysis showed a transient increase in eTregs after discontinuation in the TFR group but not in the recurrence group. This result is consistent with that of the JALSG STIM213 trial, an imatinib discontinuation trial in Japan [16], and similar results were also noted in a discontinuation trial following initial dasatinib treatment [28]. Given that Tregs suppress the immune response [29], the increase in their proportion in the TFR group appears to be paradoxical in terms of antitumor immunity. Nevertheless, an overall enhancement in antitumor immunity, including the activation of CD8^+^ T cells, was also observed. As Tregs are highly sensitive to TKIs [30], their increased proportion after TKI discontinuation could indicate a sustained host immune response, whereas the lack of change in their proportion could indicate a diminished host immune system following long‐term TKI exposure [16].

We also detected statistically significant differences in the proportions of EM and EMRA CD8^+^ T cells 1 and 3 months after TFR between the TFR and recurrence groups. Despite the occurrence of molecular relapses, including those in this study, within 6 months of TKI discontinuation, the increase in EM CD8^+^ T cells in the TFR group continued for at least 6 months in the current study. Patients with a high number of EM CD8^+^ T cells (> 29.5%) also exhibited a higher TFS 1 month after bosutinib discontinuation. CD8^+^ T cells are classically subdivided into naïve, effector, and memory T cells, according to their differentiation state [31]. Memory T cells are further classified into central memory, EM, and tissue‐resident cells [32, 33]. Generally, EM CD8^+^ T cells are a long‐lived population of immune cells that provide formidable protection against infections and malignancies and are crucial for the efficacy of vaccines and cancer immunotherapy [34].

A recent single‐cell analysis showed that the proportion of T EM/EMRA CD8^+^ T cells increased at diagnosis [35]. This subset has also been shown to express the tumor‐associated antigen PR1 and shows cytotoxic activity [35]. In this study, long‐term high EM CD8^+^ T cell levels may indicate immunity against residual tumors following TKI discontinuation. The immunological differences between the TFR and recurrent groups suggest that molecular recurrence may be suppressed by the antitumor immune response elicited in the TFR group. Although several studies have predicted the prognosis of TFR based on immune status before TKI discontinuation [14, 36, 37], no difference in immune status before TKI discontinuation was observed in this study. In contrast, the immunological changes observed in the TFR group after TKI discontinuation suggest that reactivation of the antitumor immune response, suppressed and attenuated by TKI, is crucial for long‐term TFR maintenance. Additionally, measuring T‐cell populations before and after bosutinib discontinuation may be valuable in predicting a successful TFR. Nevertheless, prospective studies with larger sample sizes involving patients who have discontinued bosutinib therapy are warranted.

## Author Contributions

**Yuki Fujioka:** conceptualization (equal), data curation (equal), formal analysis (equal), investigation (equal), methodology (equal), project administration (equal), resources (equal), software (equal), validation (equal), visualization (equal), writing – original draft (equal), writing – review and editing (equal). **Maiko Abumiya:** visualization (equal), writing – review and editing (equal). **Takaaki Ono:** data curation (equal), resources (equal), writing – review and editing (equal). **Naoto Takahashi:** conceptualization (equal), resources (equal), supervision (equal), visualization (equal), writing – review and editing (equal).

## Ethics Statement

Approval of the research protocol by an Institutional Review Board: This study was approved by the Ethics Committee of the Akita University Graduate School of Medicine (no. 2235).

## Consent

All participants provided informed consent and signed an institutional review board consent form.

## Conflicts of Interest

Naoto Takahashi received speaker fees from Novartis Pharmaceuticals and Pfizer, grants and speaker fees from Otsuka Pharmaceutical, and grants from Asahi Kasei Pharma outside of the submitted work. Takaaki Ono received speaker fees from Novartis Pharmaceuticals, Pfizer, and Otsuka Pharmaceutical, and grants from Chugai Pharma and JB Pharma outside of the submitted work. The other authors declare no conflicts of interest.

## Supporting information


**Figure S1.** Proportion and subsets of CD19^+^ B cell, CD16^+^ NK cell, and M‐MDSC. (A) Proportion of CD19^+^ B cell, (B) proportion of CD16^+^ NK cell, and (C) Proportion of M‐MDSCs in CD3‐lymphocyte before discontinuation (Pre), at 1 month (1 M), and 3 months (3 M) are presented. Significant differences can not be observed in the proportions of the cells between the TFR (blue) and recurrence (gray) groups. 1 M, 1 month after bosutinib discontinuation; 3 M, 3 months after bosutinib discontinuation; M‐MDSCs, monocytic myeloid‐derived suppressor cells; ns, not significant; Pre, immediately before bosutinib discontinuation; Rec, recurrence; TFR, treatment‐free remission.

## Data Availability

Data can be obtained from the corresponding author upon reasonable request.
