# Supplementary figures and images for: Bosutinib for Successful Treatment‐Free Remission in Chronic Myeloid Leukemia
Source: Cancer Med. 2025 Apr 28;14(9):e70822. doi: 10.1002/cam4.70822 (PMC12037706; doi:10.1002/cam4.70822)

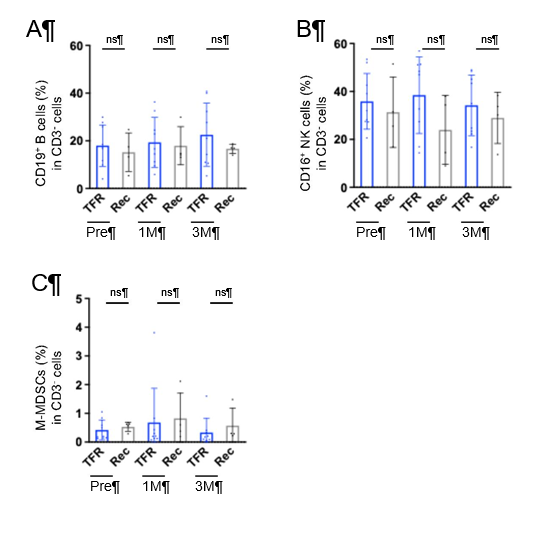

Supplement: Supplementary file 1 — Figure S1. Proportion and subsets of CD19+ B cell, CD16+ NK cell, and M‐MDSC. (A) Proportion of CD19+ B cell, (B) proportion of CD16+ NK cell, and (C) Proportion of M‐MDSCs in CD3‐lymphocyte before discontinuation (Pre), at 1 month (1 M), and 3 months (3 M) are presented. Significant differences can not be observed in the proportions of the cells between the TFR (blue) and recurrence (gray) groups. 1 M, 1 month after bosutinib discontinuation; 3 M, 3 months after bosutinib discontinuation; M‐MDSCs, monocytic myeloid‐derived suppressor cells; ns, not significant; Pre, immediately before bosutinib discontinuation; Rec, recurrence; TFR, treatment‐free remission. [file CAM4-14-e70822-s001.docx]
